# Relação Causal entre Tempo de Exibição de Televisão, Doenças Cardiovasculares e Mecanismos Potenciais

**DOI:** 10.36660/abc.20230796

**Published:** 2024-10-17

**Authors:** Mengjin Hu, Boyu Li, Jinggang Xia, Chunlin Yin, Yuejin Yang

**Affiliations:** 1 Capital Medical University Xuanwu Hospital Beijing China Xuanwu Hospital, Capital Medical University, Beijing – China; 2 State Key Laboratory of Cardiovascular Disease Fuwai Hospital Beijing China Fuwai Hospital, State Key Laboratory of Cardiovascular Disease, Beijing – China

**Keywords:** Televisão, Doenças Cardiovasculares, Fatores de Risco Cardiometabólico, Inflamação, Análise da Randomização Mendeliana

## Abstract

**Fundamento:**

Como comportamento sedentário predominante no lazer, foi documentado que assistir televisão aumenta as doenças cardiovasculares em estudos observacionais, mas a relação causal e os mecanismos potenciais ainda precisam ser determinados.

**Objetivos:**

Investigar sistematicamente a relação causal entre o tempo de exibição de televisão, doenças cardiovasculares e mecanismos potenciais.

**Métodos:**

Realizamos uma análise de randomização mendeliana (RM) de duas amostras para estimar associações causais com doenças cardiovasculares e biomarcadores de risco cardiometabólico. O método aleatório ponderado pela variância inversa foi utilizado como estimativa primária. Para contabilizar múltiplas comparações, um valor P de correção de Bonferroni para doenças cardiovasculares e biomarcadores de risco cardiometabólico foi 0,0045 e 0,0024, respectivamente.

**Resultados:**

O tempo de visualização de televisão geneticamente instrumentado foi associado a riscos mais elevados de diabetes tipo 2 (odd ratio [OR]=2,51; intervalo de confiança [IC] de 95%: 1,89-3,33; p<0,00001), hipertensão (OR=2,11; IC 95%: 1,67-2,66; p<0,00001), doença coronariana (OR=1,53; IC 95%: 1,23-1,91; p=0,00015) e insuficiência cardíaca (OR=1,42; IC 95%: 1,18-1,70; p=0,00017). Evidências sugestivas de associações prejudiciais também foram observadas para doença arterial periférica (OR=1,58; IC 95%: 1,07-2,34; p=0,02253) e acidente vascular cerebral isquêmico (OR=1,34; IC 95%: 1,10-1,63; p=0,00328). Biomarcadores de risco cardiometabólico, incluindo interleucina 10, leptina, adiposo visceral, adiposo subcutâneo abdominal, gordura hepática, índice de massa corporal, circunferência da cintura, triglicerídeos e proteína C reativa, estavam aumentados. A pressão arterial sistólica, a frequência cardíaca, a lipoproteína de baixa densidade e o colesterol total foram potencialmente aumentados, enquanto a lipoproteína de alta densidade diminuiu. No entanto, o tempo de visualização da televisão não teve efeito sobre o tromboembolismo venoso ou a embolia pulmonar.

**Conclusão:**

O tempo de exibição de televisão foi causalmente associado ao aumento do risco de doenças cardiovasculares, o que pode ser explicado por mecanismos metabólicos e inflamatórios.

## Graphical abstract

**Figure:**
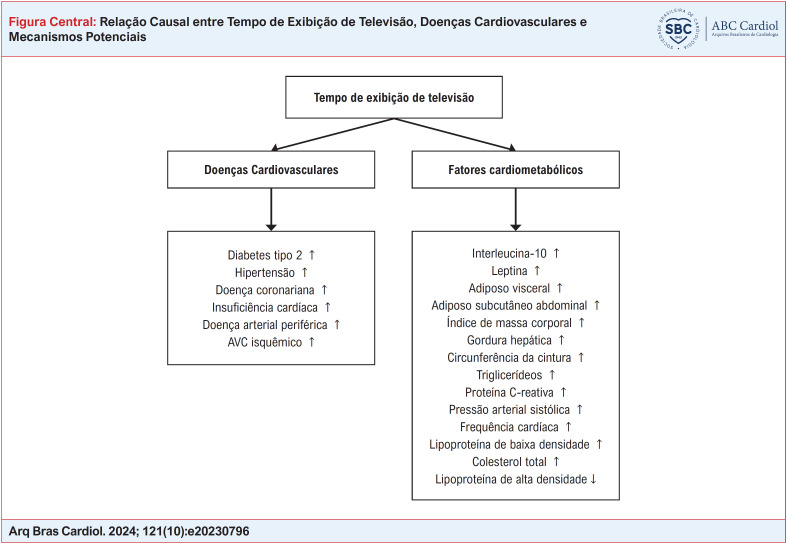
Uma visão geral do efeito do tempo de exibição de televisão sobre doenças cardiovasculares e biomarcadores de risco cardiometabólico.

## Introdução

Verificou-se que ver televisão, o comportamento sedentário predominante nos momentos de lazer em muitos países desenvolvidos, está negativamente associado a doenças cardiovasculares e a fatores de risco cardiovasculares, independentemente dos níveis de atividade física,^1 , 2^ mesmo nos adultos que são fisicamente ativos e cumprem as diretrizes de exercício.^3^ Além disso, foram documentadas relações dose-resposta, com associações moderadas para assistir televisão <2 h/d e associações mais fortes para ≥4 h/d.^4^ No entanto, vale ressaltar que a evidência de riscos mais elevados de doenças cardiovasculares é geralmente gerados a partir de estudos observacionais, que são desafiadores para interpretar a causalidade devido à existência de fatores de confusão. Embora várias variáveis potenciais de confusão tenham sido ajustadas, é provável que outros fatores de confusão não medidos ou desconhecidos, como interação em redes sociais ou solidão, possam resultar em tempo prolongado de visualização de televisão, especialmente para indivíduos mais velhos. Além disso, permanece a incerteza se o tempo prolongado de visualização de televisão ocorreu antes, durante ou após o aparecimento de doenças cardiovasculares. Embora os participantes com doenças cardiovasculares diagnosticadas relevantes tenham sido excluídos para evitar causalidade reversa, outras características, como o excesso de peso, provavelmente podem levar os participantes a passarem mais tempo frente a tela da televisão. Portanto, a causalidade reversa não pode ser descartada. Determinar as ligações causais de fatores de risco potencialmente modificáveis com doenças cardiovasculares é de grande importância para a compreensão da etiologia das doenças cardiovasculares bem como a prevenção e gestão de doenças cardiovasculares em ambientes clínicos. Na prática, ensaios clínicos randomizados (ECR) que aumentam especificamente a exposição à televisão são um método ideal para inferir causalidade. No entanto, o ECR é demorado e difícil de realizar por razões práticas ou éticas.

Atualmente, a randomização mendeliana (RM) é cada vez mais usada para examinar os efeitos causais das exposições em doenças cardiovasculares, uma vez que as variantes genéticas são determinadas na concepção e, portanto, não são afetadas por fatores de confusão ou causalidade reversa.^5^ No presente estudo, investigamos sistematicamente se o tempo de visualização de televisão geneticamente previsto está causalmente associado a doenças cardiovasculares. Além disso, os mecanismos que ligam o tempo de visualização de televisão e as doenças cardiovasculares permanecem desconhecidos, e não há evidências claras de uma relação entre o tempo de visualização de televisão e biomarcadores de risco cardiometabólico. Embora a maioria dos estudos tenha relatado associações significativas entre o tempo de exibição de televisão e a obesidade em adultos, essas associações desapareceram após ajuste para o índice de massa corporal (IMC) basal.^6^ Portanto, a associação entre o tempo de exibição de televisão e biomarcadores de risco cardiometabólico também foi investigada para encontrar potenciais mecanismos subjacentes às doenças cardiovasculares.

## Métodos

### Desenho do estudo

**Figura 1:**
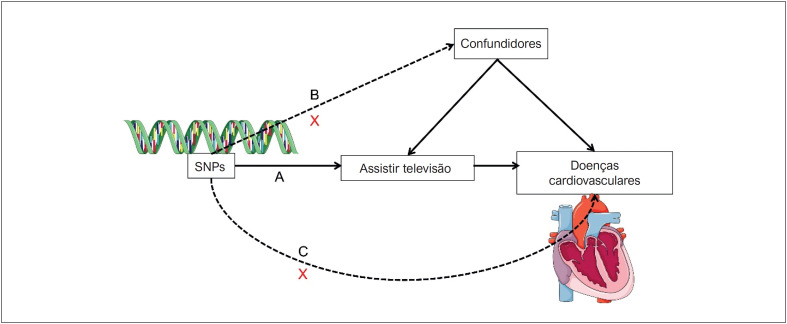
Três pressupostos principais do estudo de randomização mendeliana. Os SNPs devem estar fortemente associados ao tempo de exibição de televisão; B. Os SNPs devem ser independentes de confundidores; C. Os SNPs só devem ser associados ao risco de doenças cardiovasculares/biomarcadores através do tempo de visualização da televisão. SNP: polimorfismo de nucleotídeo único.

Os polimorfismos de nucleotídeo único (SNPs) selecionados como as variantes genéticas para o tempo de exibição de televisão tiveram que atender às três suposições a seguir: A) Os SNPs estão fortemente associados ao tempo de exibição de televisão; B) Os SNPs não estão correlacionados com confundidores conhecidos; C) Os SNPs afetam doenças cardiovasculares e biomarcadores de risco cardiometabólico apenas através do tempo de exibição de televisão ( Figura 1 ).^7^

### Fonte de dados

Os participantes da nossa análise de RM de duas amostras eram predominantemente de ascendência europeia. Estatísticas resumidas para a associação de cada SNP com o tempo de exibição de televisão foram obtidas do UK Biobank.^8^ As doenças cardiovasculares investigadas incluíram doença coronariana, hipertensão, fibrilação atrial, insuficiência cardíaca, diabetes tipo 2, acidente vascular cerebral isquêmico, ataque isquêmico transitório, tromboembolismo venoso, embolia pulmonar, doença arterial periférica e morte cardíaca. Os biomarcadores de risco cardiometabólico investigados incluíram pressão arterial sistólica (PAS), pressão arterial diastólica (PAD), frequência cardíaca, IMC, adiposo visceral, adiposo subcutâneo abdominal, gordura hepática, leptina, circunferência da cintura, proteína C reativa (PCR), Interleucina 6 (IL-6), Interleucina 10 (IL-10), adiponectina, fator transformador de crescimento-β (TGF-^β^), fator de necrose tumoral-^α^ (TNF-^α^), colesterol total, triglicerídeos, lipoproteína de alta densidade (HDL), lipoproteína de baixa densidade (LDL), glicemia de jejum e fibrinogênio. As características iniciais dos estudos de associação genômica ampla incluídos (GWAS) podem ser encontradas na Tabela Suplementar 1. A aprovação ética não foi aplicável para a presente análise porque todos os dados GWAS incluídos estão disponíveis publicamente e foram aprovados pelos conselhos de revisão ética relevantes.

### Seleção de SNP

Consideramos os SNPs atingindo significância em todo o genoma (5 × 10−8) e avaliamos a força de cada SNP usando a estatística F, com F ≥10 sendo considerado um instrumento forte. Para garantir que a contribuição dos SNPs incluídos fosse independente, o desequilíbrio de ligação foi verificado. Quando r2 > 0,001 (janela de agregação de 10.000 kb), os SNPs associados a mais SNPs ou com maior valor de p foram deletados.

### Análise de randomização mendeliana

A ponderação da variância inversa (IVW) com efeito aleatório foi considerada a principal estimativa para mitigar a influência da heterogeneidade. Várias análises de sensibilidade foram realizadas, incluindo mediana ponderada, RM-Egger, modo simples e modo ponderado. Um método de mediana ponderada pode fornecer estimativas consistentes mesmo que até 50% da informação provenha de SNPs inválidos.^9^ O método RM-Egger assume que os efeitos pleiotrópicos são independentes da distribuição de variantes genéticas associadas à exposição. Além disso, os SNPs selecionados como variantes genéticas para o tempo de exibição de televisão podem estar correlacionados com fatores de confusão. Ao verificar o intercepto do RM-Egger, podemos avaliar a pleiotropia horizontal dos SNPs selecionados.^10^ Além disso, para determinar o efeito de um SNP individual nas estimativas globais, foi realizada uma análise de sensibilidade de exclusão. O valor Q de Cochrane foi utilizado para avaliar a heterogeneidade entre os SNPs selecionados. Para contabilizar comparações múltiplas, foi utilizado um valor p de correção de Bonferroni (p corrigido: 0,05/11 = 0,0045 para doenças cardiovasculares e p corrigido: 0,05/21 = 0,0024 para biomarcadores de risco cardiometabólico). O valor de p entre o valor corrigido por Bonferroni e 0,05 sugeriu evidência de associação, sendo necessária confirmação adicional. Todas as análises estatísticas foram realizadas utilizando os pacotes "TwoSampleRM" na versão R 4.0.3 (R Foundation for Statistical Computing, Viena, Áustria).

## Resultados

### Instrumentos genéticos para tempo de exibição de televisão

Conforme mostrado na Tabela Suplementar 2, obtivemos 113 SNPs associados ao tempo de exibição de televisão e todos F>10.

### Associações com doenças cardiovasculares

**Figura 2:**
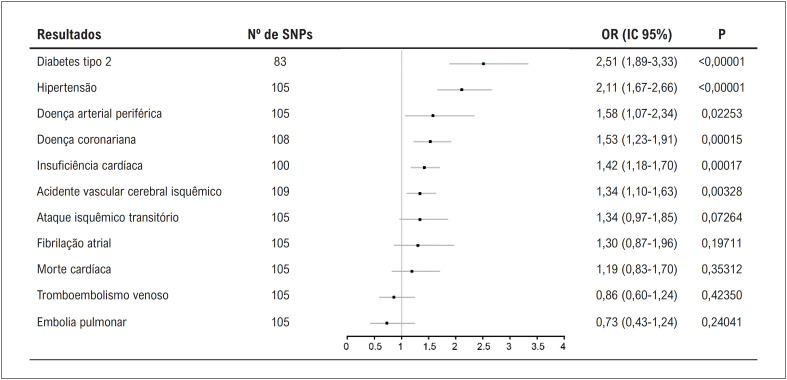
Associações do tempo de visualização de televisão geneticamente previsto com doenças cardiovasculares. IC: intervalo de confiança; OR: odd ratio; SNP: polimorfismo de nucleotídeo único.

Geneticamente instrumentado o tempo de exibição de televisão afetou negativamente quatro das 11 doenças cardiovasculares, incluindo e com magnitude decrescente de associações: diabetes tipo 2, hipertensão, doença coronariana e insuficiência cardíaca. Evidências sugestivas de associações prejudiciais também foram observadas para doença arterial periférica e acidente vascular cerebral isquêmico. Entretanto, nenhuma associação foi observada para ataque isquêmico transitório, fibrilação atrial, morte cardíaca, tromboembolismo venoso ou embolia pulmonar ( Figura 2 ). Os resultados da mediana ponderada também revelaram estimativas consistentes, embora nenhuma associação tenha sido observada nos resultados do RM-Egger ( Tabela 1 ). Nos métodos simples e ponderados, nenhuma associação foi encontrada (Tabela Suplementar 3). Como a heterogeneidade foi maior para a maioria das doenças cardiovasculares ( Tabela 1 ), a IVW sob modelo aleatório foi adotada como estimativa primária. A interceptação do RM-Egger não sugeriu nenhuma evidência de pleiotropia direcional ( Tabela 1 ), ou seja, os SNPs selecionados como variantes genéticas para o tempo de exibição de televisão não foram correlacionados com fatores de confusão.

**Tabela 1:** Associações entre o tempo de visualização de televisão previsto geneticamente e doenças cardiovasculares em análises de sensibilidade usando os métodos de mediana ponderada e MR-Egger

Desfechos	Mediana Ponderada		MR-Egger		Heterogeneidade		Pleiotropia	
	OR (IC 95%)	p	OR (IC 95%)	p	Q	p	Intercepto	p
Doença coronária	1,52 (1,17-1,97)	0,00170	1,66 (0,61-4,52)	0,32058	167	<0,01	-0,001	0,87
Hipertensão	2,00 (1,51-2,65)	<0,00001	1,23 (0,40-3,85)	0,71801	174	<0,01	0,0063	0,35
Fibrilação atrial	1,57 (0,88-2,81)	0,12969	1,19 (0,16-8,75)	0,86696	130	0,04	0,0011	0,92
Insuficiência cardíaca	1,57 (1,25-1,98)	0,00011	1,39 (0,60-3,18)	0,44250	143	<0,01	0,0003	0,96
Diabetes tipo 2	2,43 (1,86-3,18)	<0,00001	5,73 (1,15-28,57)	0,03624	250	<0,01	-0,0095	0,31
Acidente vascular cerebral isquêmico	1,41 (1,12-1,79)	0,00385	2,06 (0,85-5,01)	0,11479	164	<0,01	-0,0051	0,34
Ataque isquêmico transitório	1,13 (0,72-1,77)	0,60293	4,21 (0,87-20,38)	0,07683	115	0,21	-0,0134	0,15
Tromboembolismo venoso	0,88 (0,56-1,39)	0,59302	1,16 (0,19-7,09)	0,87435	155	<0,01	-0,0035	0,74
Embolia pulmonar	0,79 (0,42-1,47)	0,45391	2,74 (0,20-38,07)	0,45398	159	<0,01	-0,0156	0,31
Doença arterial periférica	1,52 (0,90-2,56)	0,11353	2,49 (0,36-17,29)	0,35698	131	0,04	-0,0054	0,64
Morte cardíaca	1,31 (0,78-2,20)	0,31031	1,35 (0,23-8,01)	0,73906	109	0,36	-0,0016	0,88

**Figura 3:**
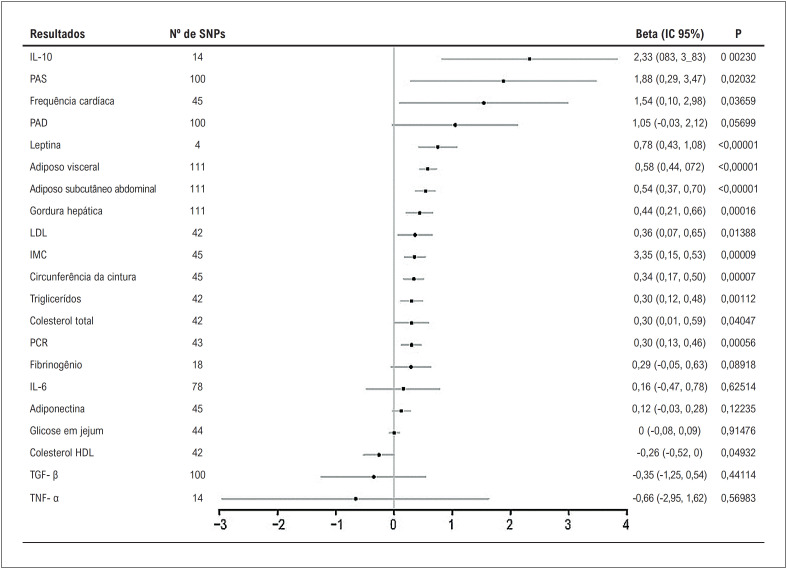
Associações do tempo de visualização de televisão geneticamente previsto com biomarcadores cardiovasculares. IMC: índice de massa corporal; IC: intervalo de confiança; PCR: proteína C reativa; PAD: pressão arterial diastólica; HDL: lipoproteína de alta densidade; IL: interleucina; LDL: lipoproteína de baixa densidade; PAS: pressão arterial sistólica; SNP: polimorfismo de nucleotídeo único; TGF: fator transformador de crescimento; TNF: fator de necrose tumoral.

Os gráficos de dispersão (Figura 1 suplementar) e gráficos de floresta (Figura 2 suplementar) da associação entre o tempo de exibição de televisão e doenças cardiovasculares documentaram resultados semelhantes. As estimativas gerais não foram afetadas desproporcionalmente por nenhum SNP individual (Figura 3 suplementar), e nenhuma evidência de pleiotropia horizontal foi observada (Figura 4 suplementar).

### Associações com biomarcadores de risco cardiometabólico

Como mostrado na Figura 3 , o tempo de exibição de televisão geneticamente instrumentado foi positivamente associado a nove dos 21 biomarcadores de risco cardiometabólico, incluindo e com magnitude decrescente de associações: IL-10, leptina, adiposo visceral, adiposo subcutâneo abdominal, gordura hepática, IMC, circunferência da cintura, triglicerídeos e PCR. Evidências sugestivas foram observadas entre o tempo de exibição de televisão geneticamente instrumentado e PAS elevada, frequência cardíaca, LDL e colesterol total, enquanto HDL baixo. Não foram encontradas associações causais significativas para PAD, fibrinogênio, IL-6, adiponectina, glicemia de jejum, TGF-^β^ ou TNF-^α^. Os resultados medianos ponderados revelaram estimativas semelhantes, enquanto apenas o HDL revelou estimativas consistentes nos resultados do RM-Egger ( Tabela 2 ). Nos métodos simples e ponderados, nenhuma associação foi encontrada (Tabela Suplementar 4). A heterogeneidade foi maior para a maioria dos biomarcadores de risco cardiometabólico ( Tabela 2 ). A evidência de pleiotropia direcional existia apenas na adiponectina, colesterol total, HDL e LDL ( Tabela 2 ).

**Tabela 2:** Associações entre o tempo de visualização de televisão previsto geneticamente e biomarcadores cardiovasculares em análises de sensibilidade usando os métodos de mediana ponderada e RM-Egger

Desfechos	Mediana Ponderada		RM-Egger		Heterogeneidade		Pleiotropia	
	Beta (IC 95%)	p	Beta (IC 95%)	p	Q	p	Intercepto	p
Pressão arterial sistólica	1,69 (0,64, 2,74)	0,00163	-4,56 (-11,48, 2,36)	0,19992	741	<0,01	0,0765	0,06
Pressão arterial diastólica	1,23 (0,63, 1,83)	0,00006	-1,91 (-6,63, 2,81)	0,42931	1026	<0,01	0,0352	0,21
Frequência cardíaca	1,10 (-0,97, 3,17)	0,29726	-2,06 (-9,65, 5,54)	0,59814	42	0,56	0,0407	0,35
Índice de massa corporal	0,22 (0,06, 0,39)	0,00686	1,17 (0,31, 2,03)	0,01098	133	<0,01	-0,0093	0,06
Gordura visceral	0,63 (0,44, 0,81)	<0,00001	0,81 (0,18, 1,44)	0,01289	149	0,01	-0,0028	0,46
Gordura subcutânea abdominal	0,48 (0,27, 0,69)	0,00001	0,65 (-0,09, 1,39)	0,08797	174	<0,01	-0,0013	0,76
Gordura hepática	0,34 (0,13, 0,54)	0,00134	0,56 (-0,46, 1,57)	0,28591	285	<0,01	-0,0014	0,81
Leptina	0,63 (-0,05, 1,32)	0,07082	2,54 (-1,40, 6,49)	0,33364	1	0,8	-0,0276	0,46
Circunferência da cintura	0,21 (0,02, 0,40)	0,03374	0,55 (-0,29, 1,39)	0,20845	85	<0,01	-0,0024	0,62
Proteína C-reativa	0,36 (0,19, 0,53)	0,00002	0,27 (-0,57, 1,10)	0,53739	104	<0,01	0,0003	0,94
Interleucina 6	-0,21 (-1,05, 0,62)	0,61895	-1,84 (-4,69, 1,02)	0,21138	88	0,18	0,0240	0,17
Interleucina 10	2,32 (-0,77, 5,42)	0,14130	6,11 (-6,85, 19,07)	0,37353	5	0,97	-0,0407	0,57
Adiponectina	0,19 (-0,01, 0,39)	0,06947	0,94 (0,17, 1,71)	0,02056	68	0,01	-0,0092	0,04
Fator de crescimento transformador β	-0,09 (-1,43, 1,26)	0,89742	-1,65 (-5,70, 2,41)	0,42775	98	0,50	0,0153	0,52
Fator de necrose tumoral α	0,26 (-3,04, 3,56)	0,87779	-0,31 (-13,35, 12,73)	0,96397	12	0,52	-0,0038	0,96
Colesterol total	0,22 (-0,04, 0,47)	0,09477	-1,80 (-3,14, −0,46)	0,01174	173	<0,01	0,0240	<0,01
Triglicerídeos	0,22 (0,02, 0,42)	0,02895	0,70 (-0,21, 1,62)	0,14147	79	<0,01	-0,0046	0,39
Lipoproteína de alta densidade	-0,17 (-0,40, 0,06)	0,14656	-2,44 (-3,58, −1,30)	0,00015	157	<0,01	0,0249	<0,01
Lipoproteína de baixa densidade	0,13 (-0,11, 0,37)	0,27773	-1,09 (-2,49, 0,31)	0,13381	161	<0,01	0,0166	0,04
Glicose em jejum	0,01 (-0,13, 0,15)	0,92310	-0,06 (-0,51, 0,39)	0,80228	38	0,68	0,0007	0,78
Fibrinogênio	0,49 (0,18, 0,79)	0,00206	0,75 (-0,23, 1,73)	0,15503	45	<0,01	-0,0062	0,35

IC: intervalo de confiança; RM: randomização mendeliana; OR: razão de chances.

Os gráficos de dispersão (Figura 5 suplementar) e gráficos de floresta (Figura 6 suplementar) da associação entre o tempo de exibição de televisão e biomarcadores de risco cardiometabólico apresentaram resultados semelhantes. Um único SNP não afetou desproporcionalmente as estimativas gerais (Figura 7 suplementar). Nenhuma evidência de pleiotropia horizontal foi encontrada nos gráficos de funil (Figura 8 suplementar).

Uma visão geral do efeito do tempo de exibição de televisão sobre doenças cardiovasculares e biomarcadores de risco cardiometabólico podem ser encontrados na Figura Central.

## Discussão

Esta análise de RM confirmou estudos observacionais anteriores, demonstrando associações causais entre o tempo de exibição de televisão e riscos aumentados de diabetes tipo 2, hipertensão, doença coronariana e insuficiência cardíaca. Confirmamos ainda a nova descoberta de que essa associação foi mediada principalmente por marcadores inflamatórios e metabólicos, incluindo aumento de IL-10,leptina, PCR, adiposo visceral, adiposo subcutâneo abdominal, gordura hepática, IMC, circunferência da cintura e triglicerídeos. PAS, frequência cardíaca, LDL e colesterol total foram potencialmente aumentados enquanto o HDL diminuiu. No entanto, o tempo de visualização da televisão não teve efeito sobre o tromboembolismo venoso ou a embolia pulmonar.

Ver televisão é um dos comportamentos sedentários comuns que envolvem ficar sentado por muito tempo. Além de dormir, ver televisão foi o comportamento que mais ocupava o tempo no ambiente doméstico. O tempo médio gasto assistindo televisão foi de cerca de 3 horas/dia na Austrália e no Reino Unido e chegou a 8 horas/dia nos Estados Unidos.^4^ Uma metanálise de estudos de coorte prospectivos sugeriu que assistir televisão aumentava os riscos do tipo 2 diabetes, doenças cardiovasculares e mortalidade por todas as causas. Existiu um aumento linear tanto para a diabetes tipo 2 como para as doenças cardiovasculares, e a associação com a mortalidade por todas as causas pareceu mais forte com o tempo de visualização de televisão >3 h/d.^2^ No entanto, embora os estudos incluídos tenham controlado vários fatores de risco conhecidos, o efeito da fatores de confusão residuais ou não medidos sobre os resultados não podem ser descartados. Embora os participantes com doença crónica no início do estudo tenham sido excluídos, a causalidade reversa ainda pode existir se os participantes com fases subclínicas da doença se tornarem mais sedentários. Por exemplo, a Coorte Britânica de Nascimentos sugeriu que a frequência de ver televisão estava positivamente associada à PCR, fibrinogénio, circunferência da cintura, PAS e PAD, independentemente dos hábitos de ver televisão e da atividade física. No entanto, essas associações atenuaram-se para nulas após o ajuste para o IMC basal.^11^ Os resultados do estudo do UK Biobank também revelaram que, embora o tempo de visualização de televisão estivesse associado tanto à doença isquêmica do coração (taxa de risco [HR] = 1,30; intervalo de confiança [IC] de 95%: 1,27-1,33) e morte acidental (HR = 1,15; 95). IC %: 1,07-1,24) em modelos não ajustados, as associações foram atenuadas e convergiram consideravelmente para doença cardíaca isquêmica (HR = 1,09, IC 95%: 1,06-1,12) e morte acidental (HR = 1,06, IC 95%: 0,98-1,15) após ajuste para fatores de confusão.^12^ Ao aplicar a análise RM, podemos superar o efeito dos fatores de confusão e da causalidade reversa. Além disso, a RM pode estabelecer marcadores de risco para doenças crónicas, uma vez que as variantes genéticas podem refletir a exposição ao longo da vida. Nós revelamos a relação causal entre o tempo prolongado de visualização de televisão e o aumento dos riscos de diabetes tipo 2, hipertensão, doença coronariana e insuficiência cardíaca. Os riscos aumentados podem ser explicados por níveis mais elevados de IL-10, leptina, adiposo visceral, adiposo subcutâneo abdominal, gordura hepática, IMC, circunferência da cintura, triglicerídeos e PCR. Concordante com nossos resultados, uma metanálise de quatro ECRs mostrou que a redução do tempo de exibição de televisão em crianças e jovens pode reduzir o IMC.^13^ Outros resultados de RM também apoiaram um efeito causal entre o tempo de exibição de televisão e doença arterial coronariana (odd ratio [OR]: 1,44; IC95%: 1,25-1,66; p<0,001),^14^ e acidente vascular cerebral isquêmico (OR: 1,28; IC95%: 1,10-1,49; p= 0,04).^15^

No entanto, pouco se sabe sobre o efeito do tempo de visualização de televisão sobre outras doenças cardiovasculares e sobre os mecanismos que podem estar subjacentes aos correlatos cardiometabólicos do comportamento de ver televisão. Demonstrar a plausibilidade biológica é essencial, pois ajuda a compreender a natureza causal de uma associação. Do ponto de vista comportamental, a visualização prolongada de televisão reduz o tempo de prática de atividades físicas, resultando na redução do gasto energético de todo o corpo. Assistir televisão foi associado ao aumento do comportamento de lanches, como maior consumo de lanches ricos em energia, bebidas açucaradas e fast food, enquanto menor consumo de frutas e vegetais. Além disso, os anúncios de salgadinhos na televisão podem atrair indivíduos a consumir lanches e bebidas com alto teor energético e podem desencadear comportamentos alimentares automáticos que são independentes da fome.^16^ De uma perspectiva fisiológica, assistir televisão geralmente ocorre após uma grande refeição noturna, quando o fígado/periférico a sensibilidade à insulina e o tráfego lipídico são subótimos, em parte devido à cronobiologia circadiana.^17^ Assistir televisão foi associado à perda da estimulação contrátil local, resultando na supressão da atividade da lipoproteína lipase (LPL) do músculo esquelético. A LPL é a enzima limitante da taxa envolvida na absorção de triglicerídeos e ácidos graxos livres no músculo esquelético e na produção de HDL. Além disso, a captação de glicose também foi reduzida através da translocação embotada dos transportadores de glicose GLUT-4 para a superfície das células musculares esqueléticas.^18^ Outra via potencial pode envolver a alteração da composição corporal, especialmente depósitos de gordura intra-abdominal, incluindo tecido adiposo visceral, tecido adiposo subcutâneo abdominal e gordura hepática, que são fatores de risco para dislipidemia, intolerância à glicose, hipertensão e doenças cardiovasculares.^19^ Em nossos resultados de RM, foram observados maior circunferência da cintura, adiposo visceral, adiposo subcutâneo abdominal, gordura hepática e IMC. O tecido adiposo é um local significativo para a produção de mediadores inflamatórios, o que pode levar a um maior risco de mortalidade relacionada à inflamação com o aumento do tempo de exibição de televisão. Stamatakis et al.^20^ sugeriram que a inflamação de baixo grau pode explicar cerca de 20% da associação entre lazer baseado em telas e eventos cardiovasculares. No tempo prolongado de visualização de televisão, aqueles com peso médio tendem a ter maior risco de mortalidade relacionada com inflamação em comparação com indivíduos com excesso de peso.^21^ Concordante com a opinião de que os biomarcadores inflamatórios, incluindo PCR, IL-10 e leptina, estavam aumentados em nossa análise de RM. Distúrbios lipídicos também foram observados em nossos resultados de RM, refletidos por triglicerídeos, LDL e colesterol total elevados, enquanto HDL baixo, que são fatores de risco conhecidos para doenças cardiovasculares. Entretanto, o tempo de exibição de televisão não afetou o tromboembolismo venoso, a embolia pulmonar ou os marcadores hemostáticos (fibrinogênio).

Comparado com outros comportamentos sedentários, ver televisão é provavelmente mais suscetível a mudanças voluntárias. Altos níveis de atividade física poderiam atenuar, mas não eliminaram, o aumento do risco de mortalidade associado ao tempo prolongado de visualização de televisão.^22^ Portanto, além da ênfase contínua na atividade física, sugestões sobre a redução do tempo de visualização de televisão podem fornecer uma mensagem clínica e de saúde pública valiosa em prevenção de doenças cardiovasculares e biomarcadores de risco cardiometabólico. A coorte ARIC (Risco de Aterosclerose nas Comunidades), baseada em 13.534 participantes, demonstrou que, em comparação com assistir mais televisão, assistir menos televisão estava associado a uma maior expectativa de vida livre de doença coronariana, acidente vascular cerebral e insuficiência cardíaca.^23^ As pausas na posição sentada podem aumentar a expressão muscular de genes envolvidos em vias anti-inflamatórias e antioxidantes (por exemplo, N-metiltransferase e cadeia leve de dineína LC8 tipo 1),^24^ e foram beneficamente associados aos níveis de triglicerídeos, IMC, circunferência da cintura e glicose plasmática de 2 horas.^25^ Portanto, as diretrizes dos EUA para crianças recomendam não mais do que duas horas de tempo de tela por dia.^26^ No entanto, os números atuais indicam que 62-83% dos adolescentes dos países ocidentais excedem as recomendações baseadas na tela.^27^ Pior ainda, as condições cardiometabólicas como a obesidade aumentam rapidamente nos EUA, afetando aproximadamente 17% de todas as crianças e adolescentes, e mais de um terço de todos os adultos.^28^ Portanto, além da atividade física, a redução do tempo de visualização de televisão deve ser visada na infância, antes de se tornar um comportamento crônico. Mais estudos são necessários para validar o papel da limitação do tempo de visualização de televisão na prevenção de doenças cardiovasculares.

### Limitações

Em primeiro lugar, as associações encontradas são relativas à população europeia e não podem ser generalizadas para outras pessoas. No entanto, estudos entre negros também revelaram que assistir >4 horas de televisão estava associado a riscos mais elevados de doenças cardiovasculares e mortalidade por todas as causas, em comparação com assistir <2 horas de televisão diariamente.^29^ A origem europeia também excluiu a influência do viés de estratificação populacional nos resultados. Em segundo lugar, a heterogeneidade foi substancial na maioria dos resultados. Portanto, foi adotado um modelo de efeitos aleatórios para mitigar a influência da heterogeneidade, e as análises de sensibilidade da mediana ponderada também produziram resultados semelhantes. Terceiro, a falta de dados brutos no GWAS original nos limita de fazer análises mais aprofundadas.

## Conclusões

Ao aplicar a análise de RM livre de fatores de confusão e causalidade reversa, nossos resultados indicaram que o tempo de exibição de televisão estava causalmente associado ao aumento dos riscos de diabetes tipo 2, hipertensão, doença coronariana e insuficiência cardíaca. Essa associação foi mediada principalmente por marcadores inflamatórios e metabólicos, incluindo aumento de IL-10, leptina, PCR, adiposo visceral, adiposo subcutâneo abdominal, gordura hepática, IMC, circunferência da cintura e triglicerídeos. No entanto, o tempo de visualização da televisão não teve efeito sobre o tromboembolismo venoso ou a embolia pulmonar. Dada a elevada prevalência de visualização excessiva de televisão, para além da ênfase contínua na atividade física, as recomendações de saúde pública deveriam considerar aconselhar uma redução do tempo de visualização de televisão.

## *Material suplementar

Para tabelas suplementares, por favor, clique aqui.
