# Supplementary material for: Relação Causal entre Tempo de Exibição de Televisão, Doenças Cardiovasculares e Mecanismos Potenciais
Source: Arq Bras Cardiol. 2024 Oct 17;121(10):e20230796. [Article in Portuguese] doi: 10.36660/abc.20230796 (PMC11634206; doi:10.36660/abc.20230796)
Supplement: Translation

# Translation: Causal Relationship between Television Viewing Time, Cardiovascular Diseases, and Potential Mechanisms

**DOI:** 10.36660/abc.20230796i

**Authors:** Mengjin Hu, Boyu Li, Jinggang Xia, Chunlin Yin, Yuejin Yang

**Affiliations:** 1 Beijing China Xuanwu Hospital, Capital Medical University, Beijing – China; 2 Beijing China Fuwai Hospital, State Key Laboratory of Cardiovascular Disease, Beijing – China

**Keywords:** Television, Cardiovascular Diseases, Cardiometabolic Risk Factors, Inflammation, Mendelian Randomization Analysis

## Abstract

**Background:**

As the predominant leisure-time sedentary behavior, television viewing was documented to increase cardiovascular diseases in observational studies, yet the causal relationship and potential mechanisms remain to be determined.

**Objectives:**

To systematically investigate the causal relationship between television viewing time, cardiovascular diseases, and potential mechanisms.

**Methods:**

We conducted a two-sample Mendelian randomization (MR) analysis to estimate causal associations with cardiovascular diseases and biomarkers of cardiometabolic risk. The random inverse-variance weighted method was used as the primary estimate. To account for multiple comparisons, a Bonferroni correction p value for cardiovascular diseases and biomarkers of cardiometabolic risk was 0.0045 and 0.0024, respectively.

**Results:**

Genetically instrumented television viewing time was associated with higher risks of type 2 diabetes (odd ratio [OR]=2.51; 95% confidence interval [CI]: 1.89-3.33; p<0.00001), hypertension (OR=2.11; 95% CI: 1.67-2.66; p<0.00001), coronary heart disease (OR=1.53; 95% CI: 1.23-1.91; p=0.00015), and heart failure (OR=1.42; 95% CI: 1.18-1.70; p=0.00017). Suggestive evidence of harmful associations was also observed for peripheral artery disease (OR=1.58; 95% CI: 1.07-2.34; p=0.02253) and ischemic stroke (OR=1.34; 95% CI: 1.10-1.63; p=0.00328). Biomarkers of cardiometabolic risk, including interleukin 10, leptin, visceral adipose, abdominal subcutaneous adipose, liver fat, body mass index, waist circumference, triglycerides, and C-reactive protein, were increased. Systolic blood pressure, heart rate, low-density lipoprotein, and total cholesterol were potentially increased while high-density lipoprotein was decreased. However, television viewing time had no effect on venous thromboembolism or pulmonary embolism.

**Conclusion:**

Television viewing time was causally associated with increased risks of cardiovascular diseases, which may be explained by metabolic and inflammatory mechanisms.

## Introduction

Television viewing, the predominant leisure-time sedentary behavior in many developed countries, was found to be detrimentally associated with cardiovascular diseases and cardiovascular risk factors independent of levels of physical activity,^1 , 2^even in those adults who are physically active and met exercise guidelines.^3^In addition, dose-response relationships have been documented, with moderate associations for television viewing <2 h/d and stronger associations for ≥4 h/d.^4^However, it is noteworthy that the evidence for higher risks of cardiovascular diseases is generally generated from observational studies, which are challenging to interpret causality due to the existence of confounding factors. Although several potential confounding variables were adjusted, it is probable that other unmeasured or unknown confounding factors, such as social network interaction or loneliness, may result in prolonged television viewing time, especially for older individuals. Moreover, uncertainty remains as to whether the prolonged television viewing time occurred before, during, or after the onset of cardiovascular diseases. Even though participants with relevant diagnosed cardiovascular diseases were excluded to avoid reverse causation, other characteristics such as being overweight may probably lead participants to spend more time on the television screen. Therefore, reverse causality cannot be ruled out. Determining the causal links of potentially modifiable risk factors with cardiovascular diseases is of great significance in understanding the etiology of cardiovascular diseases as well as in preventing and managing cardiovascular diseases in clinical settings. In practice, randomized controlled trials (RCT) specifically increasing exposure to television viewing are an ideal method to infer causality. However, RCT is time-consuming and challenging to perform for practical or ethical reasons.

Currently, Mendelian randomization (MR) is increasingly used to examine the causal effects of exposures on cardiovascular diseases as genetic variants are determined at conception and therefore are not affected by confounding factors or reverse causality.^5^In the current study, we systematically investigated whether the genetically predicted television viewing time is causally associated with cardiovascular diseases. Besides, the mechanisms linking television viewing time and cardiovascular diseases remain unknown, and there is no clear evidence of a relationship between television viewing time and biomarkers of cardiometabolic risk. Although the majority of studies reported significant associations between television viewing time and obesity in adults, these associations disappeared after adjustment for baseline body mass index (BMI).^6^Therefore, the association between television viewing time and biomarkers of cardiometabolic risk was also investigated to find potential mechanisms underlying cardiovascular diseases.

## Methods

### Study design

The single nucleotide polymorphisms (SNPs) selected as genetic variants for television viewing time had to meet the following three assumptions: A. SNPs are strongly associated with television viewing time; B. SNPs are not correlated with known confounders; C. SNPs affect cardiovascular diseases and biomarkers of cardiometabolic risk only via television viewing time ( Figure 1 ).^7^

**Figure 1:** Three key assumptions of Mendelian randomization study. SNPs must be strongly associated with television viewing time; B. SNPs must be independent of confounders; C. SNPs must only be associated with the risk of cardiovascular diseases/biomarkers via television viewing time. SNP: single nucleotide polymorphism.

### Data Source

Participants in our two-sample MR analysis were predominantly of European ancestry. Summary statistics for the association of each SNP with television viewing time were obtained from the UK Biobank.^8^The investigated cardiovascular diseases included coronary heart disease, hypertension, atrial fibrillation, heart failure, type 2 diabetes, ischemic stroke, transient ischemic attack, venous thromboembolism, pulmonary embolism, peripheral artery disease, and cardiac death. The investigated biomarkers of cardiometabolic risk included systolic blood pressure (SBP), diastolic blood pressure (DBP), heart rate, body mass index, visceral adipose, abdominal subcutaneous adipose, liver fat, leptin, waist circumference, C-reactive protein (CRP), Interleukin 6 (IL-6), Interleukin 10 (IL-10), adiponectin, transforming growth factor−β (TGF-β), tumor necrosis factor−α (TNF−α), total cholesterol, triglycerides, high-density lipoprotein (HDL), low-density lipoprotein (LDL), fasting glucose, and fibrinogen. The baseline characteristics of included genome-wide association studies (GWAS) can be found in Supplementary Table 1 . Ethics approval was not applicable for the present analysis because all included GWAS data are publicly available and had been approved by relevant ethical review boards.

**Table 1:** Associations between genetically predicted television viewing time and cardiovascular diseases in sensitivity analyses using the weighted median and MR-Egger methods

Outcomes	Weighted median		MR-Egger		Heterogeneity		Pleiotropy	
	OR (95% CI)	p	OR (95% CI)	p	Q	p	Intercept	p
Coronary heart disease	1.52 (1.17-1.97)	0.00170	1.66 (0.61-4.52)	0.32058	167	<0.01	-0.001	0.87
Hypertension	2.00 (1.51-2.65)	<0.00001	1.23 (0.40-3.85)	0.71801	174	<0.01	0.0063	0.35
Atrial fibrillation	1.57 (0.88-2.81)	0.12969	1.19 (0.16-8.75)	0.86696	130	0.04	0.0011	0.92
Heart failure	1.57 (1.25-1.98)	0.00011	1.39 (0.60-3.18)	0.44250	143	<0.01	0.0003	0.96
Type 2 diabetes	2.43 (1.86-3.18)	<0.00001	5.73 (1.15-28.57)	0.03624	250	<0.01	-0.0095	0.31
Ischemic stroke	1.41 (1.12-1.79)	0.00385	2.06 (0.85-5.01)	0.11479	164	<0.01	-0.0051	0.34
Transient ischemic attack	1.13 (0.72-1.77)	0.60293	4.21 (0.87-20.38)	0.07683	115	0.21	-0.0134	0.15
Venous thromboembolism	0.88 (0.56-1.39)	0.59302	1.16 (0.19-7.09)	0.87435	155	<0.01	-0.0035	0.74
Pulmonary embolism	0.79 (0.42-1.47)	0.45391	2.74 (0.20-38.07)	0.45398	159	<0.01	-0.0156	0.31
Peripheral artery disease	1.52 (0.90-2.56)	0.11353	2.49 (0.36-17.29)	0.35698	131	0.04	-0.0054	0.64
Cardiac death	1.31 (0.78-2.20)	0.31031	1.35 (0.23-8.01)	0.73906	109	0.36	-0.0016	0.88

CI: confidence interval; MR: Mendelian randomization; OR: odd ratio.

### SNP Selection

We considered SNPs reaching genome-wide significance (5 × 10^−8^) and evaluated the strength of each SNP using the F-statistic, with F ≥10 being considered a strong instrument. To ensure the contribution of included SNPs was independent, linkage disequilibrium was checked. When r^2^> 0.001 (clumping window 10000 kb), the SNP associated with more SNPs or with a higher p value were deleted.

### MR analysis

Inverse variance weighting (IVW) with random effect was regarded as the main estimate to mitigate the influence of heterogeneity. Several sensitivity analyses were conducted, including weighted median, MR-Egger, simple mode, and weighted mode. A weighted median method can give consistent estimates even if up to 50% of the information is from invalid SNPs.^9^MR-Egger method assumes that the pleiotropic effects are independent of the distribution of genetic variants associated with the exposure. Moreover, the SNPs selected as genetic variants for television viewing time may be correlated with confounders. By checking the intercept from MR-Egger, we can evaluate the horizontal pleiotropy of selected SNPs.^10^Additionally, to determine the effect of an individual SNP on the overall estimates, a leave-one-out sensitivity analysis was performed. Cochrane's Q value was used to assess heterogeneity among selected SNPs. To account for multiple comparisons, a Bonferroni correction p value (corrected p: 0.05/11 = 0.0045 for cardiovascular diseases and corrected p: 0.05/21 = 0.0024 for biomarkers of cardiometabolic risk) was used. P value between the Bonferroni-corrected value and 0.05 suggested evidence of association, and further confirmation was required. All statistical analyses were conducted using the "TwoSampleMR" packages in R version 4.0.3 (R Foundation for Statistical Computing, Vienna, Austria).

## Results

### Genetic instruments for television viewing time

As shown in Supplementary Table 2 , we obtained 113 SNPs associated with television viewing time and all F >10.

**Table 2:** Associations between genetically predicted television viewing time and cardiovascular biomarkers in sensitivity analyses using the weighted median and MR-Egger methods

Outcomes	Weighted median		RM-Egger		Heterogeneity		Pleiotropy	
	Beta (95% CI)	p	Beta (95% CI)	p	Q	p	Intercept	p
Systolic blood pressure	1.69 (0.64. 2.74)	0.00163	-4.56 (-11.48. 2.36)	0.19992	741	<0.01	0.0765	0.06
Diastolic blood pressure	1.23 (0.63. 1.83)	0.00006	-1.91 (-6.63. 2.81)	0.42931	1026	<0.01	0.0352	0.21
Heart rate	1.10 (-0.97. 3.17)	0.29726	-2.06 (-9.65. 5.54)	0.59814	42	0.56	0.0407	0.35
Body mass index	0.22 (0.06. 0.39)	0.00686	1.17 (0.31. 2.03)	0.01098	133	<0.01	-0.0093	0.06
Visceral adipose	0.63 (0.44. 0.81)	<0.00001	0.81 (0.18. 1.44)	0.01289	149	0.01	-0.0028	0.46
Abdominal subcutaneous adipose	0.48 (0.27. 0.69)	0.00001	0.65 (-0.09. 1.39)	0.08797	174	<0.01	-0.0013	0.76
Liver fat	0.34 (0.13. 0.54)	0.00134	0.56 (-0.46. 1.57)	0.28591	285	<0.01	-0.0014	0.81
Leptin	0.63 (-0.05. 1.32)	0.07082	2.54 (-1.40. 6.49)	0.33364	1	0.8	-0.0276	0.46
Waist circumference	0.21 (0.02. 0.40)	0.03374	0.55 (-0.29. 1.39)	0.20845	85	<0.01	-0.0024	0.62
C-Reactive protein	0.36 (0.19. 0.53)	0.00002	0.27 (-0.57. 1.10)	0.53739	104	<0.01	0.0003	0.94
Interleukin 6	-0.21 (-1.05. 0.62)	0.61895	-1.84 (-4.69. 1.02)	0.21138	88	0.18	0.0240	0.17
Interleukin 10	2.32 (-0.77. 5.42)	0.14130	6.11 (-6.85. 19.07)	0.37353	5	0.97	-0.0407	0.57
Adiponectin	0.19 (-0.01. 0.39)	0.06947	0.94 (0.17. 1.71)	0.02056	68	0.01	-0.0092	0.04
Transforming growth factor-β	-0.09 (-1.43. 1.26)	0.89742	-1.65 (-5.70. 2.41)	0.42775	98	0.50	0.0153	0.52
Tumor necrosis factor-α	0.26 (-3.04. 3.56)	0.87779	-0.31 (-13.35. 12.73)	0.96397	12	0.52	-0.0038	0.96
Total cholesterol	0.22 (-0.04. 0.47)	0.09477	-1.80 (-3.14. −0.46)	0.01174	173	<0.01	0.0240	<0.01
Triglycerides	0.22 (0.02. 0.42)	0.02895	0.70 (-0.21. 1.62)	0.14147	79	<0.01	-0.0046	0.39
High-density lipoprotein	-0.17 (-0.40. 0.06)	0.14656	-2.44 (-3.58. −1.30)	0.00015	157	<0.01	0.0249	<0.01
Low-density lipoprotein	0.13 (-0.11. 0.37)	0.27773	-1.09 (-2.49. 0.31)	0.13381	161	<0.01	0.0166	0.04
Fasting glucose	0.01 (-0.13. 0.15)	0.92310	-0.06 (-0.51. 0.39)	0.80228	38	0.68	0.0007	0.78
Fibrinogen	0.49 (0.18. 0.79)	0.00206	0.75 (-0.23. 1.73)	0.15503	45	<0.01	-0.0062	0.35

CI: confidence interval; MR: Mendelian randomization; OR: odd ratio.

### Associations with cardiovascular diseases

Genetically instrumented television viewing time detrimentally affected four of the 11 cardiovascular diseases, including and with decreasing magnitude of associations: type 2 diabetes, hypertension, coronary heart disease, and heart failure. Suggestive evidence of harmful associations was also observed for peripheral artery disease and ischemic stroke. However, no association was observed for transient ischemic attack, atrial fibrillation, cardiac death, venous thromboembolism, or pulmonary embolism ( Figure 2 ). The weighted median results also revealed consistent estimates, while no association was observed in MR-Egger results ( Table 1 ). In simple and weighted mode methods, no association was found (Supplementary Table 3). As heterogeneity was higher for the majority of cardiovascular diseases ( Table 1 ), IVW under a random model was adopted as the primary estimate. The intercept from MR-Egger suggested no evidence of directional pleiotropy ( Table 1 ), that is the SNPs selected as genetic variants for television viewing time were not correlated with confounders.

**Figure 2:** Associations of genetically predicted television viewing time with cardiovascular diseases. CI: confidence interval; OR: odd ratio; SNP: single nucleotide polymorphism.

The scatter plots (Supplementary Figure 1) and forest plots (Supplementary Figure 2) of the association between television viewing time and cardiovascular diseases documented similar results. The overall estimates were not disproportionately affected by any individual SNP (Supplementary Figure 3), and no evidence of horizontal pleiotropy was observed (Supplementary Figure 4).

### Associations with biomarkers of cardiometabolic risk

As shown in Figure 3 , genetically instrumented television viewing time was positively associated with nine of the 21 biomarkers of cardiometabolic risk, including and with decreasing magnitude of associations: IL-10, leptin, visceral adipose, abdominal subcutaneous adipose, liver fat, BMI, waist circumference, triglycerides, and CRP. Suggestive evidence was observed between genetically instrumented television viewing time and high SBP, heart rate, LDL, and total cholesterol while low HDL. No significant causal associations were found for DBP, fibrinogen, IL-6, adiponectin, fasting glucose, TGF-β or TNF-α. The weighted median results revealed similar estimates, while just HDL revealed consistent estimates in MR-Egger results ( Table 2 ). In simple and weighted mode methods, no association was found (Supplementary Table 4). The heterogeneity was higher for the majority of biomarkers of cardiometabolic risk ( Table 2 ). The evidence of directional pleiotropy just existed in adiponectin, total cholesterol, HDL, and LDL ( Table 2 ).

**Figure 3:** Associations of genetically predicted television viewing time with cardiovascular biomarkers. BMI: body mass index; CI: confidence interval; CRP: C-Reactive protein; DBP: diastolic blood pressure; HDL: high-density lipoprotein; IL: interleukin; LDL: low-density lipoprotein; SBP: systolic blood pressure; SNP: single nucleotide polymorphism; TGF: transforming growth factor; TNF: tumor necrosis factor.

The scatter plots (Supplementary Figure 5) and forest plots (Supplementary Figure 6) of the association between television viewing time and biomarkers of cardiometabolic risk showed similar results. A single SNP did not disproportionately affect the overall estimates (Supplementary Figure 7). No evidence of horizontal pleiotropy was found in the funnel plots (Supplementary Figure 8).

An overview of the effect of television viewing time on cardiovascular diseases and biomarkers of cardiometabolic risk can be found in the Central Illustration.

## Discussion

This MR analysis confirmed previous observational studies by demonstrating causal associations between television viewing time and increased risks of type 2 diabetes, hypertension, coronary heart disease, and heart failure. We further confirmed the novel finding that this association was mainly mediated by inflammatory and metabolic markers, including increased IL-10, leptin, CRP, visceral adipose, abdominal subcutaneous adipose, liver fat, BMI, waist circumference, and triglycerides. SBP, heart rate, LDL, and total cholesterol were potentially increased while HDL was decreased. However, television viewing time had no effect on venous thromboembolism or pulmonary embolism.

Television viewing is one of the common sedentary behaviors that involve prolonged sitting. Aside from sleeping, television viewing was the behavior that occupied the most time in the domestic setting. The average time spent watching television was about 3 h/d in both Australia and the United Kingdom and was up to 8 h/d in the United States.^4^A meta-analysis of prospective cohort studies suggested that television viewing increased the risks of type 2 diabetes, cardiovascular disease, and all-cause mortality. A linear increase existed for both type 2 diabetes and cardiovascular disease, and the association with all-cause mortality appeared stronger with television viewing time >3 h/d.^2^However, although the included studies controlled for various known risk factors, the effect of residual or unmeasured confounding factors on outcomes cannot be ruled out. Although participants with chronic disease at baseline were excluded, reverse causality may still exist if participants with subclinical stages of disease become more sedentary. For example, the British Birth Cohort suggested that television viewing frequency was positively associated with CRP, fibrinogen, waist circumference, SBP, and DBP independent of television viewing habits and physical activity. However, these associations attenuated towards null after adjusting for baseline BMI.^11^Results from the UK Biobank study also revealed that although television viewing time was associated with both ischemic heart disease (hazard ratio [HR]=1.30; 95% confidence interval [CI]: 1.27-1.33) and accidental death (HR=1.15; 95% CI: 1.07-1.24) in unadjusted models, the associations were attenuated and considerably converged for ischaemic heart disease (HR=1.09, 95% CI: 1.06-1.12) and accidental death (HR=1.06, 95% CI: 0.98-1.15) after adjustment for confounders.^12^By applying MR analysis, we can overcome the effect of confounding factors and reverse causality. Moreover, MR can establish the risk markers for chronic disease as genetic variants can reflect life-long exposure. We revealed the causal relationship between prolonged television viewing time and increased risks of type 2 diabetes, hypertension, coronary heart disease, and heart failure. The increased risks may be explained by higher IL-10, leptin, visceral adipose, abdominal subcutaneous adipose, liver fat, BMI, waist circumference, triglycerides, and CRP. Concordant with our results, a meta-analysis of four RCTs showed that reducing television viewing time in children and youth can reduce BMI.^13^Other MR results also supported a causal effect between television viewing time, coronary artery disease (odd ratio [OR]: 1.44; 95%CI: 1.25-1.66; p<0.001),^14^and ischemic stroke (OR: 1.28; 95%CI: 1.10-1.49; p=0.04).^15^

However, less is known about the effect of television viewing time on other cardiovascular diseases and the mechanisms that might underlie the cardiometabolic correlates of television viewing behavior. Demonstrating biological plausibility is essential as it helps understand the causal nature of an association. From a behavioral perspective, prolonged television viewing reduces time engaging in physical activities, resulting in reduced whole-body energy expenditure. Television viewing was associated with increased snacking behavior, such as higher intakes of energy-dense snacks, sugar-sweetened beverages, and fast foods while lower intakes of fruits and vegetables. Moreover, snack-food advertisements on television may attract individuals to consume high-energy snacks and beverages and can trigger automatic eating behaviors that are independent of hunger.^16^From a physiological perspective, television viewing commonly occurs after a large evening meal when liver/peripheral insulin sensitivity and lipid trafficking are suboptimal, partly because of circadian chronobiology.^17^Television viewing was associated with loss of local contractile stimulation, resulting in the suppression of skeletal muscle lipoprotein lipase (LPL) activity. LPL is the rate-limiting enzyme involved in the uptakes of triglycerides and free fatty acids into skeletal muscle and the production of HDL. In addition, glucose uptake was also reduced through blunted translocation of GLUT-4 glucose transporters to the skeletal muscle cell surface.^18^Another potential pathway may involve the changed body composition, especially intra-abdominal fat depots, including visceral adipose tissue, abdominal subcutaneous adipose, and liver fat, which are risk factors for dyslipidemia, glucose intolerance, hypertension, and cardiovascular disease.^19^In our MR results, higher waist circumference, visceral adipose, abdominal subcutaneous adipose, liver fat, and BMI were observed. Adipose tissue is a significant site to produce inflammatory mediators, which may lead to a higher risk of inflammatory-related mortality with increasing television viewing time. Stamatakis et al.^20^suggested that low-grade inflammation may explain about 20% of the association between screen-based leisure and cardiovascular events. In prolonged television viewing time, those with average weight tended to be at greater risk of inflammatory-related mortality compared with overweight individuals.^21^Concordant with the opinion, that the inflammatory biomarkers, including CRP, IL-10, and leptin, were increased in our MR analysis. Lipid disorders were also observed in our MR results as reflected by high triglycerides, LDL, and total cholesterol while low HDL, which are known risk factors for cardiovascular diseases. However, television viewing time did not affect venous thromboembolism, pulmonary embolism, or hemostatic markers (fibrinogen).

Compared with other sedentary behaviors, television viewing is probably most susceptible to voluntary change. High levels of physical activity could attenuate but did not eliminate the increased mortality risk associated with prolonged television viewing time.^22^Therefore, apart from the continued emphasis on physical activity, suggestions regarding reducing television viewing time may provide a valuable clinical and public health message in preventing cardiovascular diseases and biomarkers of cardiometabolic risk. The ARIC (Atherosclerosis Risk in Communities) cohort based on 13534 participants demonstrated that compared with watching more television, watching less television was associated with longer life expectancy free of coronary heart disease, stroke, and heart failure.^23^Breaks in sitting could increase muscle expression of genes involved in anti-inflammatory and anti-oxidative pathways (e.g., N-methyltransferase and dynein light chain LC8-type 1),^24^and were beneficially associated with the levels of triglycerides, BMI, waist circumference, and 2-h plasma glucose.^25^Therefore, the USA guidelines for children recommend no more than two hours of screen time per day.^26^However, current figures indicated that 62-83% of adolescents from Western countries exceed the screen-based recommendations.^27^Worse still, cardiometabolic conditions such as obesity increase rapidly in the USA, affecting approximately 17% of all children and adolescents, and more than one-third of all adults.^28^Therefore, apart from physical activity, reducing television viewing time should be targeted in childhood, before it becomes a chronic behavior. Further studies are required to validate the role of limiting television viewing time in the prevention of cardiovascular diseases.

### Limitations

First, the associations found are relative to the European population, and may not generalize to others. However, studies among blacks also revealed that watching >4 hours of television was associated with higher risks of cardiovascular diseases and all-cause mortality compared with watching <2 hours of television daily.^29^European origin also excluded the influence of population stratification bias on results. Second, the heterogeneity was substantial in the majority of outcomes. Therefore, a random-effects model was adopted to mitigate the influence of heterogeneity, and sensitivity analyses of the weighted median also yielded similar results. Third, the lack of raw data in the original GWAS limits us from making further analyses.

## Conclusions

By applying MR analysis free from confounding factors and reverse causality, our results indicated that television viewing time was causally associated with increased risks of type 2 diabetes, hypertension, coronary heart disease, and heart failure. This association was mainly mediated by inflammatory and metabolic markers, including increased IL-10, leptin, CRP, visceral adipose, abdominal subcutaneous adipose, liver fat, BMI, waist circumference, and triglycerides. However, television viewing time had no effect on venous thromboembolism or pulmonary embolism. Given the high prevalence of excessive television viewing, apart from the continued emphasis on physical activity, public health recommendations should consider advising a reduction in television viewing time.

## *Supplemental Materials

For supplementary tables, please click here.
